# Mitochondrial isolation: when size matters

**DOI:** 10.12688/wellcomeopenres.16300.2

**Published:** 2020-12-02

**Authors:** Alexander G. Bury, Amy E. Vincent, Doug M. Turnbull, Paolo Actis, Gavin Hudson

**Affiliations:** 1 Wellcome Trust Centre for Mitochondrial Research, Medical School, Newcastle University, Newcastle-upon-Tyne, NE1 7RU, UK; 2 Biosciences Institute, Medical School, Newcastle University, Newcastle-upon-Tyne, NE1 7RU, UK; 3 Pollard Institute, School of Electronic and Electrical Engineering, University of Leeds, Leeds, LS2 9JT, UK; 4 Translational and Clinical Research Institute, Medical School, Newcastle University, Newcastle-upon-Tyne, NE1 7RU, UK

**Keywords:** Mitochondria, mtDNA, mitochondrial isolation, heterogeneity, subcellular, nanoprobes, nanobiopsy, nanotweezers.

## Abstract

Mitochondrial vitality is critical to cellular function, with mitochondrial dysfunction linked to a growing number of human diseases. Tissue and cellular heterogeneity, in terms of genetics, dynamics and function means that increasingly mitochondrial research is conducted at the single cell level. Whilst there are several technologies that are currently available for single-cell analysis, each with their advantages, they cannot be easily adapted to study mitochondria with subcellular resolution. Here we review the current techniques and strategies for mitochondrial isolation, critically discussing each technology’s limitations for future mitochondrial research. Finally, we highlight and discuss the recent breakthroughs in sub-cellular isolation techniques, with a particular focus on nanotechnologies that enable the isolation of mitochondria from subcellular compartments. This allows isolation of mitochondria with unprecedented spatial precision with minimal disruption to mitochondria and their immediate cellular environment.

## Introduction

### Mitochondrial genetics

The mitochondrial genome consists of multiple double-stranded, circular DNA molecules ( Anderson *et al*., 1981; Andrews *et al*., 1999). In healthy individuals the default state of the mitochondrial genome is that of homoplasmy, where only wild-type mitochondrial DNA (mtDNA) exists. Low levels of mtDNA heterogeneity as a result of *de novo* mutations, termed heteroplasmy, is not deleterious since the polyploid nature of the mitochondrial genome buffers low levels of heteroplasmy ( Payne *et al*., 2013). However, clonal expansion of mutant mtDNA above a threshold level, results in biochemical defects and disease ( Payne *et al*., 2013; Rossignol *et al*., 2003). Over a life-course it is believed that clonal expansion of mtDNA point mutations occurs through random genetic drift in both germline and mitotic somatic cells ( Greaves *et al*., 2014); as well as in post-mitotic cells, facilitated by relaxed mtDNA replication, which can explain the long-time required for a threshold level to be reached as observed in age-related disease ( Elson *et al*., 2001). In comparison, it is suggested that some degree of selection influences clonal expansion of mtDNA deletions, acting in conjunction or independently of random genetic drift ( Lawless *et al*., 2020). Asides from random genetic drift, two alternative theories of clonal expansion of mtDNA deletions are the ‘negative feedback loop’ and ‘perinuclear niche’ theories ( Kowald & Kirkwood, 2014; Vincent *et al.*, 2018). The non-uniform nature of mtDNA deletions suggests deletion of a single locus, leading to impaired feedback of polymerase γ activity, is unlikely to be solely responsible for clonal expansion of mtDNA deletions ( Damas *et al*., 2014), whilst the contribution of retrograde signalling within the ‘perinculear theory’ requires further investigation ( Lawless *et al*., 2020). To elucidate the mechanism of clonal expansion of mtDNA mutations, further investigation of mtDNA heterogeneity at the tissue, single-cell and subcellular level is necessary ( Lawless *et al*., 2020). 

### Mitochondrial disease and heterogeneity

Clonally expanded mtDNA mutations cause mitochondrial disease in one of two forms: primary, due to inherited mtDNA mutations and acquired, due to nuclear DNA mutations that lead to mtDNA mutation formation throughout life, as a result of impaired mtDNA maintenance ( Alston *et al*., 2017; Trifunovic *et al*., 2004). Primary mitochondrial disease, caused by inherited mtDNA mutations, has a variable age of onset and has a range of different presentations ( Gorman *et al*., 2016). In contrast, acquired mitochondrial disease, resulting from clonal expansion of sporadic mtDNA mutations, typically begins in adult life and causes progressive external ophthalmoplegia often with additional myopathic and neurodegenerative symptoms ( Alston *et al*., 2017); or neurodegeneration associated with advanced age ( Bender *et al*., 2006; Trifunovic *et al*., 2004).

Mitochondrial disease often expresses mosaicism in oxidative phosphorylation (OXPHOS) deficiency at the organ and tissue level, caused by cellular heterogeneity in mtDNA ( Ahmed *et al*., 2018). Mitochondrial heterogeneity exists as either genetic or non-genetic heterogeneity ( Aryaman *et al*., 2019). Sources of genetic heterogeneity associated with mitochondrial disease include mtDNA copy number, which is depleted in OXPHOS deficient skeletal muscle fibres ( Lehmann *et al*., 2019) and in Parkinson’s disease (PD) substantia nigra (SN) neurons but elevated in aged control SN neurons, possibly indicative of a neuroprotective mechanism that is overwhelmed in PD ( Chen *et al*., 2020; Lehmann *et al*., 2019). Genetic heterogeneity is also observed in elevated intercellular heteroplasmy, corresponding with oxidative phosphorylation (OXPHOS) deficiency between skeletal muscle fibres, colonic crypt cells and PD SN neurons ( Lehmann *et al*., 2019; Greaves *et al*., 2014 and Bender *et al*., 2006) but also localised intracellular heteroplasmy within skeletal muscle fibres and PD SN neurons ( Reeve *et al*., 2018; Vincent *et al*., 2018).

Non-genetic heterogeneity is observed in morphology, membrane potential and dynamics ( Kuznetsov & Margreiter, 2009). Mitochondrial heterogeneity can be physiological, due to different subcellular roles of mitochondrial subpopulations. In skeletal muscle, intermyofibrillar mitochondria have a more complex structure, likely to facilitate the contractile movement of muscle fibres ( Kuznetsov & Margreiter, 2009; Vincent *et al*., 2019). In contrast, subsarcolemmal mitochondria are more punctate mitochondria and have a lower capacity for OXPHOS ( Lai *et al*., 2019), but are likely to be involved in mito-nuclear signalling ( Paszkiewicz *et al*., 2017). It is also suggested synaptic mitochondria subpopulations have different proteomic profiles compared with non-synaptic mitochondria, notably in complex I expression, resulting in a smaller, punctate mitochondrial morphology and may modulate ATP supply for synaptic transmission and Ca ^2+^ buffering ( Graham *et al*., 2017). This proteomic heterogeneity, however, may also make synaptic mitochondria more vulnerable to OXPHOS deficiency and increased synaptic mitochondria density may serve as a compensatory mechanism, as observed in neurodegenerative disease ( Graham *et al*., 2017; Reeve *et al*., 2018). Membrane potential (Ψm) also expresses intercellular heterogeneity based on specific cell functions, such as in pancreatic β cells in response to elevated glucose and ATPase activity. Highly metabolic cells also tend to have a higher Ψm and likewise cellular regions with variable metabolic demands express localised Ψm heterogeneity ( Wikstrom *et al*., 2009). In neurodegeneration, it has been proposed that dysfunctional, depolarised mitochondria are selectively transported towards the cell body for degradation, whilst healthy mitochondria populate areas of greatest ATP demand ( Miller & Sheetz, 2004). Genetic heterogeneity is suggested to impact on non-genetic heterogeneity and that mtDNA might locally modulate cristae remodelling and respiratory chain complex structure ( Busch *et al*., 2014), equally non-genetic factors may affect mtDNA heterogeneity through mitochondrial dynamics and Ψm depolarisation modulating selective degradation of mitochondria harbouring high levels of heteroplasmy ( Wikstrom *et al*., 2009). Precise sampling of mitochondria from within subcellular localisations, to supplement existing data from studies at the single-cell and tissue level, would provide insight not only into mtDNA heterogeneity but also how this influences heterogeneity in mitochondrial structure, function and dynamics in health and disease.

### Studying mitochondrial subpopulations

There is still much we do not understand about mitochondrial heterogeneity. Investigating mitochondrial heterogeneity at the inter- and intracellular level, will provide insight into other key outstanding questions within the mitochondrial field ( Aryaman *et al*., 2019; Lawless *et al*., 2020). What is the physiological role of intracellular heterogeneity? What differences exist between inherited and acquired mtDNA mutations? What interplay exists between genetic and non-genetic heterogeneity? Historically, mitochondrial fractionation techniques were used and remain popular due to being well characterised and reliable; despite being laborious, resource intensive and potentially damaging to mitochondria ( Liao *et al*., 2020). When selecting a method of mitochondrial isolation, in addition to mitochondrial purity, function and yield; efficiency, downstream analyses and sample should be considered. Whilst cell lines are more readily manipulatable and enable longitudinal studies ( Lawless *et al*., 2020), disease burden is better characterised in tissue, which also has greater clinical relevance ( Ahmed *et al*., 2018; Chen *et al*., 2020; Grady *et al*., 2018; Spinazzi *et al*., 2012). In tissue, some foci of deficiency measure as little as 10µm in diameter ( Vincent *et al*., 2018) and accurate sampling of individual foci would be challenging using current techniques. The purpose of this review is to critically discuss the advancement and application of different techniques for mitochondrial isolation. This review will also highlight the recent development of micro- and nanoscale techniques, allowing isolation of mitochondria at the cellular and subcellular level.

## Macroscale mitochondrial isolation

Macroscale mitochondrial isolation entails the release of mitochondria from organs or large tissue samples through physical disruption. This is achieved through manual homogenisation, although low reproducibility and the degree of skill required affect mitochondrial integrity and yield of mitochondria ( Lai *et al*., 2019). Subcellular fractionation, through homogenisation followed by centrifugation, is a traditional and well-established means of reliably acquiring a high-yield mitochondrial fraction. To overcome some of the noted limitations of centrifugation-based methods of mitochondrial isolation, a number of alternative macroscale techniques have been developed for use with, or instead of, differential or density gradient centrifugation ( Gauthier & Lazure, 2014).

### Centrifugation

Differential centrifugation (DC) and density gradient centrifugation (DGC) are used to fractionate organelles, including mitochondria, based on mass and sedimentary characteristics. Cell or tissue homogenate is centrifuged at increasing velocities and, in DGC, through a series of increasingly dense media bands ( Liao *et al*., 2020). DGC produces a purer fraction but a lower mitochondrial yield, relative to DC, though yield and mitochondrial integrity may be improved using detergents and reducing the homogenisation and centrifugation speed ( Lai *et al*., 2019). These methods of ‘mitochondrial fractionation’ are both reliable, acquire highly functional mitochondria and are well characterised in the literature but have a high resource and time cost. Dependent on the nature of the study, using DC alone may be preferable in obtaining a higher yield despite sacrificing mitochondrial purity. Centrifugation methods do remain the most popular methods of large-scale mitochondrial isolation however post-centrifugation methods are seeing an increase in usage, such as affinity purification ( Liao *et al*., 2020).

### Affinity purification

Affinity purification (AP) captures mitochondria, through the adhesion of mitochondrial surface proteins ( Ru *et al*., 2012), to magnetic bead-conjugated antibodies which are retained within a magnetic field. In addition to favourable yield and purity, AP allows isolation of mitochondrial subpopulations, even without centrifugation ( Ahier *et al*., 2018; Hornig-Do *et al*., 2009; Hubbard *et al*., 2019) and has a low sample requirement and run time ( Ru *et al*., 2012). AP does have a high reagent cost and is less well established than DC or DGC but with continued development it is suggested that AP could succeed DGC as the preeminent method of large-scale mitochondrial isolation and AP is particularly advantageous for isolating mitochondrial subpopulations from homogenate ( Afanasyeva *et al*., 2018; Hubbard *et al*., 2019).

### Flourescence activated organelle sorting

Following detection of a fluorescent label, including dyes or reporter genes ( Ashley *et al*., 2005; Cohen & Fox, 2001; Floros *et al*., 2018), fluorescence activated organelle sorting (FAOS) is able to isolate mitochondria from other organelles or mitochondrial subpopulations by assigning mitochondria, separated into individual droplets, a specific charge ( Cavelier *et al*., 2000). FAOS enables isolation of a higher yield of functional, purified mitochondrial subpopulations reducing the starting material requirement ( Daniele *et al*., 2016; Gauthier & Lazure, 2014). A consideration when using FAOS is dye cytotoxicity, however, the increasing number of available dyes should allow substitution of potentially toxic dyes ( Barteneva *et al*., 2014); nevertheless, fluorescent dyes may still lead to mitochondrial aggregation and overestimation of mtDNA copy number ( Pflugradt *et al*., 2011). FAOS is best utilised for high-throughput and high-yield mitochondrial isolation from larger samples ( Poe *et al.*, 2006).

### Electrophoresis and field-flow fractionation

Capillary electrophoresis (CE), free-flow electrophoresis (FFE) and field-flow fractionation (FFF) techniques also separate subpopulations of organelles based on sedimentary characteristics. Samples are carried along a channel by a laminar flow and in CE and FFE, organelles are separated based on their isoelectric point, through application of an electric field ( Poe *et al.*, 2006; Zischka *et al.*, 2006), whilst FFF utilises a perpendicular cross flow to separate particles based on size and mass ( Yang *et al*., 2015). FFE is shown to be more time efficient and able to produce a purer but smaller yield, however, equipment cost was higher compared with DGC ( Hartwig *et al*., 2009). There is consensus in the literature that FFE is optimally used in conjunction with DGC and for use in studies where high-throughput and mitochondrial fraction purity are more desirable than a higher mitochondrial yield ( Islinger *et al*., 2018; Zischka *et al*., 2006). Compared with FFE, CE-LIF (capillary electrophoresis -laser-induced fluorescence) has a lower throughput but requires less starting material, and has a better signal-to-noise ratio compared with FAOS ( Kostal *et al*., 2009; Poe *et al*., 2006). A limitation of CE-LIF is mitochondria retention within the fine capillary tube, which can impact on yield and, like FFE, is best used when purity is preferable to high yield ( Poe *et al*., 2010). FFF has the highest working range of the techniques reviewed and does not require mitochondrial labelling ( Kang *et al*., 2008; Reschiglian *et al*., 2005; Yang *et al*., 2015). Whilst isolation of mitochondrial subpopulations was demonstrated with FFF, isolated fractions were highly contaminated and with no direct comparison of yield relative to DGC, it is difficult to determine the practicability of FFF for mitochondrial isolation. Unlike FFE and CE, FFF is best used when rapid acquisition of a high mitochondrial yield is required at the expense of purity ( Kang *et al*., 2008).

### Overview of macroscale mitochondrial isolation techniques

Macroscale methods, incorporating centrifugation, remain the gold standard for high throughput, large-scale mitochondrial isolation but are limited by high time and resource demands, coupled with limited separation of mitochondrial subpopulations ( Lai *et al*., 2019; Liao *et al*., 2020) ( Table 1). Combining DGC or DC with post-fractionation purification, isolation of purified mitochondrial subpopulations is possible but at the risk of increasing the run time, sacrificing purity or impacting on mitochondrial vitality or function. Additionally, these techniques cannot provide information on cellular provenance or allow longitudinal studies, due to the necessity of cell lysis. Whilst macroscale techniques excel at investigating mitochondrial heterogenity at the tissue level, to study patterns of mitochondrial heterogeneity and dysfunction at the cellular and sub-cellular level, low-throughput analysis of smaller samples is likely to be more appropriate ( Picard *et al*., 2011).

**Table 1.** Summary of mitochondrial isolation methods (Macroscale). A table highlighting the relative benefits and disadvantages of different ‘macroscale’ mitochondrial methods of mitochondrial isolation, incorporating either density gradient centrifugation (DGC), differential centrifugation (DC) or none. Techniques compared relative to the performance of DGC alone*: ++ = higher; + = slightly higher or similar; - = lower; 2D-PAGE = two-dimensional polyacrylamide gel electrophoresis; AP = affinity purification; CE = capillary electrophoresis; FAOS = fluorescence activated organelle sorting; FFE = free flow electrophoresis; FFF = field-flow fractionation; LC-MS = liquid chromatography mass spectrometry; SEM = Scanning Electron Microscopy; TEM = transmission electron microscopy.

Isolation Method	Prior Fractionation	Mitochondrial Yield *	Mitochondrial Purity *	Starting Material *	Expense *	Throughput *	Subcellular Spatial Precision	Serial Sampling	Confirmation	General Comments	References
AP	DC (or none)	++ (++)	+ (++)	- (-)	++ (++)	++ (++)	No	No	Western blotting, immunofluorescence, qPCR	High selectivity; sonication may impact mitochondria viability; not suitable for larger samples	Hornig-Do *et al.* (2009); Ahier *et al.* (2018) and Hubbard *et al.* (2019)
FAOS	DC	++	++	++	+	++	No	No	qPCR, NGS	High selectivity; shear damage and dyes may impact mitochondrial viability	Daniele *et al.* (2016)
FFE	DC (and DGC)	+ (-)	++ (++)	+ (+)	++ (++)	++ (++)	No	No	Western blotting, TEM	High mitochondrial viability; high selectivity	Zischka *et al.* (2006)
FFF	DC	++	-	+	++	++	No	No	Western blotting, SEM, 2D-PAGE, LC-MS	High mitochondrial viability; high selectivity; high working range	Kang *et al.* (2008) and Yang *et al.* (2015)
CE	DC	-	++	-	++	++	No	No	qPCR	High selectivity; predisposed to clogging	Poe *et al.* (2006) and Poe *et al.* (2010)

## Microscale and nanoscale mitochondrial isolation

Whilst mitochondrial heterogeneity does exist at the single and subcellular level, until relatively recently there has been a distinct lack of techniques capable of effective subcellular manipulation. Previous methods enabling mechanical penetration of cells relied on micropipettes which are inclined to cause physical trauma to the cell and lack the spatio-temporal control required for subcellular sampling. Use of microfluidics and nanoprobes offer a less damaging and more controlled means of sampling of organelles such as mitochondria ( Kang *et al*., 2016) ( Table 2 and Table 3).

**Table 2.** Summary of mitochondrial isolation methods (Microscale). A table highlighting the relative benefits and disadvantages of different ‘microscale’ mitochondrial methods of mitochondrial isolation, incorporating either density gradient centrifugation (DGC), differential centrifugation (DC) or none. Techniques compared relative to the performance of DGC alone*: ++ = higher; + = slightly higher or similar; - = lower; ddPCR = digital droplet PCR; LCM = laser capture microdissection; NGS = next generation sequencing; OT = optical tweezers; SEM = Scanning Electron Microscopy.

Isolation Method	Prior Fractionation	Mitochondrial Yield *	Mitochondrial Purity *	Starting Material *	Expense *	Throughput *	Subcellular Spatial Precision	Serial Sampling	Confirmation	General Comments	References
Micro-AP	DC	-	++	-	-	++	No	No	fluorescence microscopy	High mitochondrial viability; low run time; predisposed to clogging	Kayo *et al*. (2013) and Banik *et al*. (2016)
Micro-FFE	DC	-	++	-	-	++	No	No	electrophoretic profiling	High mitochondrial viability; low run time; predisposed to clogging	Kostal *et al*. (2009)
Micro-FFF	DC	-	++	-	-	++	No	No	fluorescence microscopy	High mitochondrial viability; low run time	Lu *et al*. (2004)
LCM	None	-	++	-	++	-	Yes	No	fluorescence microscopy, ddPCR, qPCR	Targets a single cell; may cause laser induced mtDNA damage	Vincent *et al*. (2018) and Trifunov *et al*. (2018)
OT	None	-	++	-	++	-	Yes	No	fluorescence microscopy; SEM	Targets a single cell; may cause laser induced mtDNA damage	König *et al*. (2005) and Jeffries *et al*. (2007)

**Table 3.** Summary of mitochondrial isolation methods (Nanoscale). A table highlighting the relative benefits and disadvantages of different methods of nanoscale mitochondrial isolation, incorporating either density gradient centrifugation (DGC), differential centrifugation (DC) or none. Techniques compared relative to the performance of DGC alone *: ++ = higher; + = slightly higher or similar; - = lower; nFAMS = Nano-Fluorescence Activated Mitochondrial Sorting; NGS = next generation sequencing; NT = nanotweezers MS = Mass spectrometry.

Isolation Method	Prior Fractionation	Mitochondrial Yield *	Mitochondrial Purity *	Starting Material *	Expense *	Throughput *	Subcellular Spatial Precision	Fluorescent labelling	Serial Sampling	Confirmation	General Comments	References
nFAMS	DGC	++	++	-	+	++	No	Yes	No	fluorescence microscopy, MS, qPCR	High selectivity; shear damage may impact mitochondrial viability; dyes may induce aggregation	MacDonald *et al*. (2019)
Nanobiopsy	None	-	++	-	++	-	Yes	Yes	Yes	fluorescence microscopy, NGS, SICM	Maintains cellular environment and viability; limited selectivity	Actis *et al.* (2014a)
NT	None	-	++	-	++	-	Yes	Yes	Yes	fluorescence microscopy	Maintains cell viability and natural environment; limited selectivity	Nadappuram *et al*. (2019)

### Microfluidics

Microfluidic analogues of macro-techniques, including micro-AP, micro-FFF, and micro-FFE, have been developed for the isolation of mitochondria ( Kostal *et al*., 2009; Kayo *et al*., 2013; Lu *et al*., 2004). Microfluidic isolation methods also exist that trap mitochondria through geometric traps, such as the nanohole array ( Kumar *et al*., 2015). Not only are microfluidic assays more resource efficient, they are less disruptive to the physiological organelle environment, cause less physical trauma to cells and organelles and provide more representative results ( Kang *et al*., 2016; Moutaux *et al*., 2018). Whilst **‘** Mito-magento’, a micro-AP variant, forgoes the need for immunoprecipitation and minimises the risk of clogging, which can impact on yield, nanoparticle toxicity to cells and mitochondria is not ruled out ( Banik *et al*., 2016; Tesauro *et al*., 2017; Yarjanli *et al*., 2017). Microfluidic techniques have a much lower yield and do not allow insight into the subcellular location of mitochondrial subpopulations or facilitate longitudinal analysis. Microfluidic techniques are best suited to high-throughput isolation of pure and highly functional mitochondrial fractions from a limited amount of starting material.

### Nano-fluorescence activated mitochondrial sorting

FAOS is generally optimised to isolate mitochondria from larger samples ( Daniele *et al*., 2016). Nano-fluorescence activated mitochondrial sorting (nFAMS) utilises a microfluidic chip device with confocal microscopy. Application of a small voltage allows sorting of mitochondrial subpopulations with minimal impact to viability whilst achieving a high throughput, yield and purity ( MacDonald *et al.*, 2019; Schiro *et al*., 2012). It is noted single molecule PCR may be more applicable to enrichment of smaller mtDNA concentrations, obtained using nFAMS and similar formats, to avoid PCR errors ( Kraytsberg *et al*., 2008; MacDonald *et al*., 2019). Respiratory function assays did show that isolated mitochondria were highly functional, however, like the other techniques discussed so far, obligatory cell lysis also means that obtaining information on cell provenance and serial sampling are not possible with nFAMS ( MacDonald *et al*., 2019).

### Laser-capture microdissection and optical tweezers

Laser capture microdissection (LCM) allows for single-cell isolation by cutting around a delinated cell using a ‘train’ of sequential nanosecond pulses from a nitrogen laser. Translocation and capture is either gravity assisted or through laser pulse induced propulsion into a collection vessel ( Vogel *et al.*, 2007). LCM was used to isolate mitochondria from single cells and from subcellular foci, to investigate the spread of clonal expansion of mtDNA mutations a ( Trifunov *et al*., 2018; Vincent *et al*., 2018).

Optical tweezers (OT) are capable of simultaneously lysing cells, whilst trapping subcellular organelles within a focused laser beam, with nanoscale precision ( König *et al*., 2005; Reiner *et al*., 2010). LCM and OT are exciting techniques for isolating mitochondria within a specific cell, demonstrating not only the means to retain cellular provenance but also demonstrating cellular and subcellular precision ( König *et al*., 2005; Vincent *et al.*, 2018). However, laser induced damage to mitochondrial and mtDNA damage when targeting smaller areas limits the effectiveness of LCM and OT in serial sampling and isolating mitochondria from smaller subcellular foci ( Botchway *et al*., 2010; Vincent *et al.*, 2018). Alongside flow cytometry based methods, LCM and OT are gold standard techniques used for mitochondrial isolation for understanding mitochondrial heterogeneity at the single-cell level.

### Nanoprobe based techniques

Nanotechnologies show great promise to enable mitochondrial isolation at the subcellular level ( Laforge *et al.*, 2007). Whilst micromanipulators incorporating micropipettes have been shown to successfully isolate and transplant nuclei ( Hyslop *et al*., 2016), a challenge for this technology has been the ability to sample smaller subcellular structures ( Jeffries *et al*., 2007). Nanoscale probes can allow the manipulation of biomolecules and organelles in a non-invasive manner with greater spatial precision compared to their microscale counterparts. Nanoprobes cause minimal perturbation to cell viability or their natural cellular environment, allowing for longitudinal sampling of mitochondria and enabling the comparison of mitochondrial heterogeneity between and within foci of deficiency ( Actis, 2018). Four key methods for nanomanipulation of cytoplasmic contents have been developed: nanobiopsy, where cellular contents are aspirated within a nanopipette using an applied voltage; fluid-force microscopy (FluidFM), where an atomic force microscope (AFM) probe, containing a nanofluidic channel, enables pressure-driven sampling from living cells; dielectrophoretic ****nanoprobes, where an applied high-frequency AC is used to trap and manipulate organelles and biomolecules within living cells; and nanoelectroporation, where cells are grown on an array of channels and a transient electrical current causes temporary pore formation in the cellular membrane, allowing sampling from the cytoplasm ( Ino *et al*., 2018). So far only nanobiopsy and nanotweezers (NT), which harnesses dielectrophoresis, have been demonstrated to isolate mitochondria ( Actis *et al*., 2014a; Nadappuram *et al*., 2019).

**Figure 1.**
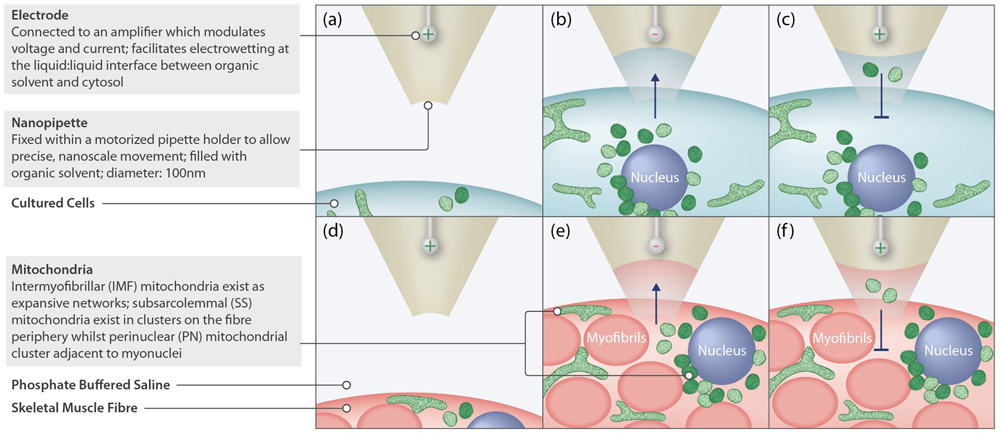
Mitochondrial nanobiopsy. Nanobiopsy is carried out using a nanopipette, filled with organic solvent, that is incorporated into a Scanning Ion Conductance Microscope (SICM) system which consists of an amplifier, that modulates and detects extremely small changes in current, as well as piezo motors that allow for highly precise movement of the nanopipette to and within the area of interest. This can be used with cultured cells ( **a**– **c**) or in tissue ( **d**– **f**). ( **a**, **d**) To function, the SICM requires the nanopipette to be immersed in an aqueous solution. Whilst being lowered, the SICM system constantly measures the current through the nanopipette tip. If the current magnitudes drops below a predetermined threshold, this will result in negative feedback causing the nanopipette to stop typically within 1µm of the cell of interest. At this point the nanopipette be lowered at high speed to penetrate the cell membrane, whilst a small positive voltage (200mV) is applied to prevent premature aspiration of the cytosolic components. ( **b**, **e**) Once within the cell and at the area of interest, application of a small voltage (-200mV) allows for the aspiration of mitochondria within the cytosol as a result of the phenomenon of electrowetting. ( **c**, **f**) After successful aspiration of mitochondria, reapplication of a positive voltage (100mV) prevents further aspiration but allows retention of the collected sample. The sample can then be transferred to a collection vessel where the sample can be expelled at a higher voltage (1V).

***Nanobiopsy*.** Nanopipettes are nanoprobes that are readily and reproducibly fabricated from glass capillaries using a laser puller ( Laforge *et al.*, 2007). Nanopipettes can be integrated within nanomanipulators to comprise a scanning ion conductance microscope (SICM), which allows for the high resolution topographical mapping of living cells and tissues ( Novak *et al*., 2009). The nanobiopsy approach relies on electrowetting, within a nanopipette, to extract cytoplasmic material from living cells. Electrowetting is a physical effect where the wetting, or interaction of a liquid with another surface, across two interfaces, can be controlled via an externally applied voltage. If a nanopipette is filled with an organic solution and immersed in an aqueous solution a liquid-liquid interface is formed at the nanopipette tip ( Laforge *et al.*, 2007). Depending on the voltage applied, aqueous solution can be drawn or expelled from the nanopipette ( Dale & Unwin, 2008). Electrowetting within the cytoplasm of a living cell allows cytosol and organelles to be aspirated ( Actis *et al*., 2014a; Figure 1). Successful aspiration of mitochondria has been reported using nanobiopsy. This was demonstrated by the presence of MitoTracker green, a mitochondrial matrix specific fluorescent dye, in the nanopipette tip, a reduction in fluorescence in the biopsied area and successful sequencing of mtDNA acquired from the biopsy ( Actis *et al*., 2014a). Dependent on the target cell and the pipette geometry, fine tuning of the electrowetting settings is necessary ( Actis *et al*., 2014b; Seger *et al*., 2012). The precise volume aspirated or expelled can be determined, and therefore controlled, by measuring the nanopipette resistance during nanobiopsy. A drawback of nanobiopsy is that the use of an organic solvent within the nanopipette does not allow the acquisition of topographical images because of the low signal-noise ratio. To be able to properly take advantage of the fine placement of the nanopipette, a double barrel pipette - filled with aqueous and organic solutions respectively, would be required so that scanning and electrowetting could be performed simultaneously ( Seger *et al*., 2012). For biopsies taken from tissue, a possible alternative could be either the pre- or post-biopsy topographical scanning of the area of interest. Nanobiopsy is highly optimised for the highly-precise isolation of small mitochondrial subpopulations either from subcellular foci or for longitudinal sampling of mitochondria from the same cell. Whilst this has been demonstrated in cultured cells this has yet to be demonstrated in tissue.

**Figure 2.**
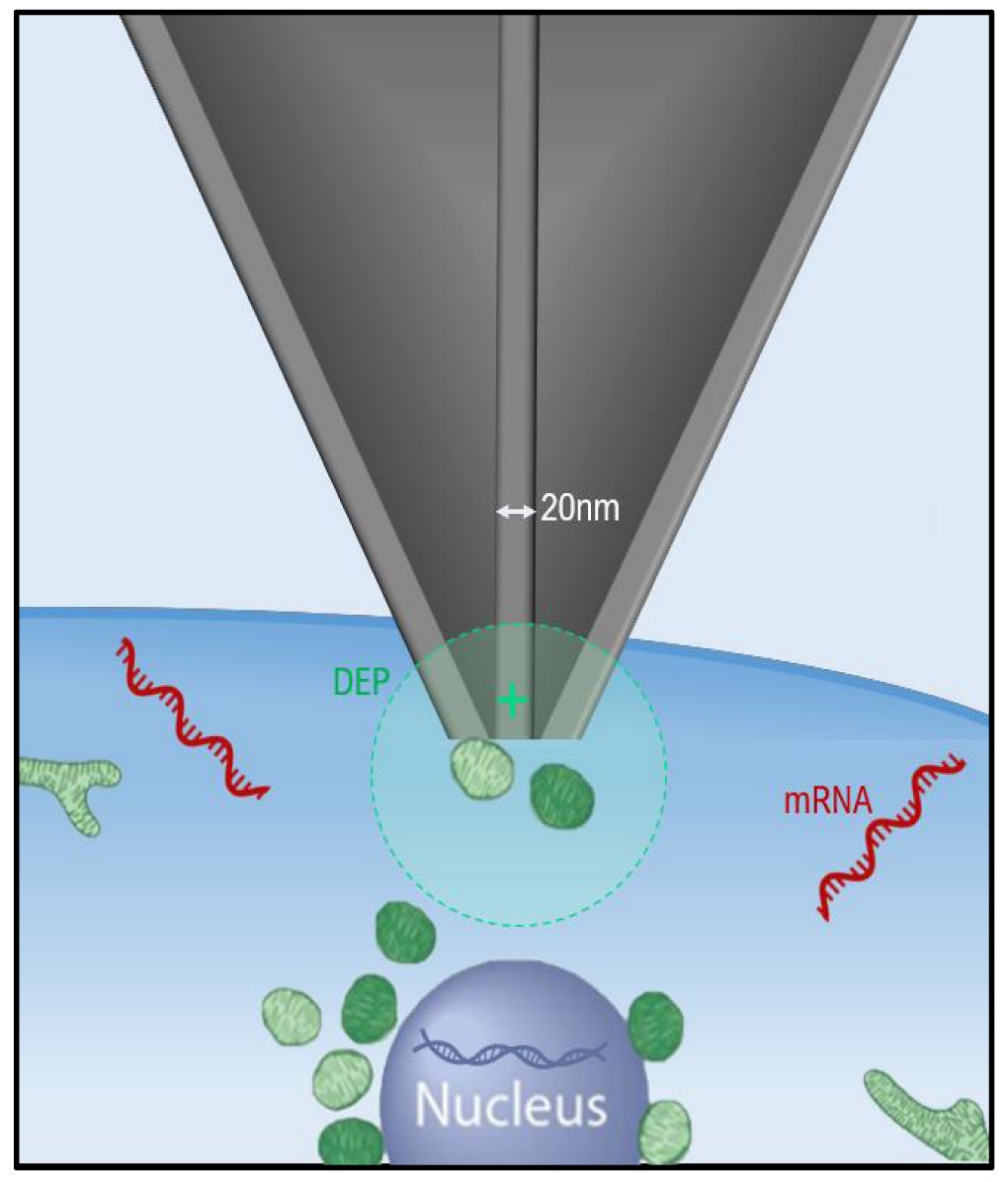
Nanotweezers. The nanotweezers tip is inserted into a cell and then application of an alternating current (A.C.) results in a localised electric field that traps biomolecules at the probe tip through dielectrophoretic (DEP). This DEP force is strong enough to capture nucleic acids and mitochondria in solution. Once captured the molecules can then we withdrawn from out of the cell of interest with the removal of the nanotweezers and transferred to a collection vessel. The applied DEP force is reversible and turning off the A.C. results in the release of the captured biomolecules.

***Nanotweezers*.** Dielectrophoretic nanotweezers (DENT) were first introduced as an adaption of AFM, where by employing dielectrophoresis, mRNA could be selectively targeted and captured with minimal impact to cell viability. DENT utilises a nanoprobe consisting of a closely distanced silicon core and a metal alloy layer which serve as inner and outer electrodes ( Nawarathna *et al*., 2009). Application of an AC between the inner and outer electrodes results in an electrical field sufficient to prompt polarisable subcellular molecules to become induced dipoles that are trapped at the probe tip ( Ramos *et al*., 1999). A dielectrophoretic force can be intensified by increasing the voltage or decreasing the distance between the two electrodes, since the force is proportionate to *V ^2^d ^−3^* ( *V* being voltage; *d* being distance). An inter-electrode distance of <20nm enabled authors to achieve a dielectrophoretic force sufficient to capture a single mitochondrion at a physiologically viable ionic strength, with a NT probe incorporated into a micromanipulator system. Successful acquisition of healthy mitochondria was shown through a reduction in localised tetramethylrhodamine methyl ester fluorescent dye post-acquisition ( Nadappuram *et al*., 2019; Figure 2). Like nanobiopsy, NT are capable of sampling subcellular biomolecules with high spatial resolution with minimal impact on cell and organelle viability. A potential disadvantage of the NT format could be the manner in which samples are retained at the end of the nanotweezers probe, similar to a holding pipette. Without protection from the pipette tip, the sample would be exposed and prone to physical trauma or loss when being transferred from the cell to collection vessel ( Imura *et al.*, 2002; Nadappuram *et al.*, 2019). Loss of nucleic acid samples can be circumvented through use of specific oligonucleotide probes at the tip of the nanotweezers, to hybridised and trap mRNA or DNA isolated through dielectrophoresis and leading to the suggestion that this technique has a greater propensity for mRNA isolation or even cell-free mtDNA, though nanotweezers still offer great promise for mitochondrial isolation ( Nadappuram *et al.*, 2019; Nawarathna *et al.*, 2009).

***Fluid-Force Microscopy*.** FluidFM is another adaptation of AFM. AFM works using a cantilever, which moves relative to the changes in depth of a given surface either through direct contact, tapping or oscillating just above the surface ( Verbiest & Rost, 2016). The movement of the cantilever is measured by a laser which produces a 3-D image of the surface and negative feedback control of a piezo motor allows adjustment of the probe position ( Guillaume-Gentil *et al*., 2016; Uehara *et al*., 2004). The unique feature of FluidFM is that it utilizes a hollow AFM tip coupled with a pressure regulator that allows the manipulation of liquid in and around cells with high precision. During FluidFM, the AFM tip is lowered into the cell and held with a predetermined force (~550nN) to allow capture of cellular contents by applying negative pressure (-800mbar) ( Guillaume-Gentil *et al*., 2016). Whilst Guillaume-Gentil and colleagues did test whether mitochondria could be isolated using FluidFM, a negligible reduction in MitoTracker Green likely indicates that this was unsuccessful. Authors concede that lack of success could have been because of probe aperture size was insufficient to capture mitochondria within an interconnected network. Like nanobiopsy and NT, FluidFM minimally impacts on cell viability. Also, like nanobiopsy, AFM allows precise placement of the AFM probe in a cellular localisation, though not with the same precision afforded by SICM. A ‘snap’ phenomenon, caused by the intermolecular forces acting on the AFM probe as it approaches the cellular membrane, can cause loss of control of the probe, reducing sampling accuracy ( Uehara *et al*., 2004; Verbiest & Rost, 2016). FluidFM also prioritises high sampling resolution above yield and should successful isolation of mitochondria be demonstrated using Fluid FM, it would fit into a similar niche to nanobiopsy and NT isolating mitochondria within subcellular foci.

### Overview of micro- and nanoscale mitochondrial isolation techniques

Microfluidic techniques are promising, being high-throughput and the microfluidic devices themselves are inexpensive. However, adapting existing platforms for microfluidics is less cost effective and has resulted in slower development and adoption of microfluidic techniques ( Sonker *et al.*, 2017). Of the more developed micro- and nanoscale techniques, flow cytometry based methods appear quicker, higher throughput and demonstrate the greatest yield, though may impair mitochondrial viability ( Pflugradt *et al*., 2011). Whilst LCM and OT can potentially result in photodamage to mtDNA, careful consideration of cutting parameters can negate this ( Keloth *et al*., 2018). Some macro- and microscale techniques may be able to separate mitochondrial subpopulations but lack the spatio-temporal range to isolate and contextualise mitochondrial subpopulations in their natural cellular environment, which affects our ability to fully understand the cellular conditions that contribute to mitochondrial heterogeneity and dysfunction ( Lanza & Nair, 2009; Moutaux *et al*., 2018).

Nanoprobes allow a means of isolating mitochondria with superior sampling specificity whilst being minimally invasive to the cell – preserving the viability of live cells and the native environment of the mitochondria ( Actis *et al.*, 2014a; Nadappuram *et al.*, 2019). It is suggested that nanobiopsy lacks specificty in the cytoplasmic contents sampled ( Actis *et al*., 2014a; Nadappuram *et al*., 2019). However, nanoscale adjustment of the nanopipette position above the area of interest, using an SICM system guided by topographical scanning in addition to optical confirmation alone, enables highly precise movement and mitochondrial capture allowing for a superior sampling specificity using nanobiopsy ( Seger *et al.*, 2012). ‘Microbiospy’ of mitochondria *in vivo* has been demonstrated using a micropipette incorporated into a micromanipulator ( Morris *et al*., 2017). Whilst the data from this study is certainly valuable, the method was limited by poor control of aspirated volumes and the inability to contextualise labelled mitochondria without a secondary mode of visualising the location of the pipette relative to labelled mitochondria. This indeed is a common limitation of both NT and other micromanipulator based methods of mitochondrial isolation. Mitochondria can be as large 2µm in diameter ( Imura *et al*., 2002). Incorporation of micropipettes within an SICM system may enable the positioning of the pipette to the mitochondrial sub-population of interest with the same nanoscale level of precision, achieved through the combination of topographical scanning and optical visualisaton, but the larger pore size would guard against sampling bias favouring smaller mitochondrial subpopulations.

NT preferentially captures polarisable molecules. Whilst this enables a degree of selectivity, this may also lead to increased contamination, especially from non-specific capture of nucleic acids ( Nadappuram *et al.*, 2019). Use of a ‘physiologically safe’ current, stated as being less than the total current of all ion channels in a given cell, also prevents direct damage to the sampled mitochondria ( Laforge *et al.*, 2007). Whilst not necessarily a disadvantage, nanobiopsy and NT both require samples to be immersed in ionic solution ( Actis, 2018). FluidFM, conversely, can be adapted to faciliate high-throughput sampling by connecting the AFM probe to a microchannel and can be carried out in or out of solution ( Kang *et al.*, 2016; Meister *et al.*, 2009). Whilst as of yet, there has only been a modest number of examples demonstrating isolation of mitochondria using nanotechnologies, pioneering proof-of-principle study data does make the future development of nanotechnologies for mitochondrial isolation an exciting prospect. Whilst sampling of mitochondria, from within foci of deficiency in tissue, has yet to be demonstrated this provides the next challenge for nanotechnologies going forward.

## Concluding remarks

Mitochondrial isolation can be organised into three general categories based on their sampling resolution: macroscale, microscale and nanoscale. Macroscale and microfluidic techniques are most useful for respective large or small-scale studies investigating mitochondrial heterogeneity, or a particular mechanism in mitochondrial dysfunction, at the tissue and organ level ( Brand & Nicholls, 2011); LCM, OT and nanoprobe based techniques, however, are designed to acertain information on mitochondrial heterogeneity at the cellular and subceullar level. The latter will ultimately help us to understand how mitochondrial dysfunction may originate and spread ( Picard *et al.*, 2011; Vincent & Picard, 2018). Whilst mitochondrial isolation has only been demonstrated using nanobiopsy and NT, asides FluidFM, other nanoprobe techniques have potential utility for mitochondrial isolation. The “mille-feuille” probe, like its namesake, contains alternating aqueous and organic phase layers, allowing continuous sampling through nano-electrophoresis ( Ito *et al.*, 2017). Nanoneedles and nanostraws offer the potential for longitudinal analysis of mitochondria from cells, cultured directly on top of these nanostructures, and sampling is achieved through laser-induced poration or electroporation of the cell membrane and high throughput isolation with a similar level of precision ( Cao *et al.*, 2019; Chiappini *et al*., 2015).

The usefulness of nanoprobe techniques is not limited to isolating mitochondria. The ability to aspirate and inject using nanoprobes has great therapeutic potential, including the injection of mitochondria at precise cellular locations for the purposes of experimentation but also the eventual possibility for use in mitochondrial targeted therapies, building upon existing mitochondrial transfer techniques whilst avoiding ‘mitochondrial carryover’ ( Hudson *et al.*, 2019; Hyslop *et al.*, 2016; Seger *et al.*, 2012). Mitochondrial fractionationation based techniques are still the most extensively used, but other technologies are catching up in terms of popularity and development. This is in part driven by the need to better understand mitochondrial physiology and pathophysiology with subcellular precision ( Picard *et al.*, 2011), but also coincides with the transition of cellular biology from era of ‘single-cell omics’ to that of ‘subceullar omics’. No single technique is all-encompassing, in aiding our understanding of mechanisms of mitochondrial dysfunction, instead each has objective superiority. Nanoprobe based technologies are a great addition to the arsenal of mitochondrial isolation methods and their continued development is opening up an entirely new avenue of research into the spread of mitochondrial dysfunction at the subcellular level. This will ultimately help us better understand the bigger picture of mitochondrial heterogeneity and dysfunction.

## Data availability

### Underlying data

No data are associated with this article.
