# Supplementary material for: Mitochondrial isolation: when size matters
Source: Wellcome Open Res. 2020 Dec 2;5:226. Originally published 2020 Oct 1. [Version 2] doi: 10.12688/wellcomeopenres.16300.2 (PMC7931255; doi:10.12688/wellcomeopenres.16300.2)
Supplement: Peer review history

# Peer review: Reviewer response for version 2

**DOI:** 10.21956/wellcomeopenres.18122.r42634

**Published:** 2021-03-03

**Authors:** Alexander Galkin

**Affiliations:** 1 Department of Pediatrics, Columbia University, New York, NY, USA

This is a very well-written manuscript that presents a scope of mitochondrial isolation techniques. In the mini-review “Mitochondrial isolation: when size matters” the authors performed a comprehensive analysis of past and current technologies for mitochondria isolation focusing on the recent advances in micro and nanoscale isolation procedures. This is a timely review on a very important topic. I have no major conceptual criticism, but the following points should be clarified: 

1. In the table, on pages 6-8 multiple abbreviations should be avoided for the sake of clarity. There is plenty of space to include a full description of the procedure (columns one and two). Other columns containing only plus and minus signs can be significantly decreased in width. This would be beneficial for a reader who might not be familiar with a new system of abbreviations.
2. In the table’s “confirmation” column several approaches for validating successful isolation are mentioned, however, there is no mentioning of cytochrome spectra or activity/respiration measurements. These methods are still widely used and represent the golden standard for confirming the quality of the preparation.
3. It is clear that Figures 1 and 2 show the principal schematics of the experimental setup of nanobiopsies and nanotweezers. However, for a better display, it would be good to put things to the scale. For example, in Fig 2 nanotweezers are schematically shown. The end tip diameter is labeled as 60-100 nm and the nucleus is roughly the same size on the picture, while in reality, it is ~5000 nm. Mitochondria are 200-400 nm in diameter but shown as smaller than 60nm.
4. The authors only briefly mentioned two references when discussing centrifugation. A more detailed analysis would significantly increase the interest in that minireview. There is a huge number of experimental papers, protocols, and reviews on the topic of differential centrifugation and this method is still one of the most commonly used and accepted in the field. Alternatively, a reference to an appropriate book or Methods in Enzymology volume can be given.

This reviewer cannot comment on the methodology of nanoscale isolation. In my opinion, the scope of the present manuscript fits very well with the audience of the Wellcome Open Research, and it can be accepted for indexing.

Is the review written in accessible language?

Yes

Are all factual statements correct and adequately supported by citations?

Yes

Are the conclusions drawn appropriate in the context of the current research literature?

Yes

Is the topic of the review discussed comprehensively in the context of the current literature?

Partly

Reviewer Expertise:

Mitochondrial physiology

I confirm that I have read this submission and believe that I have an appropriate level of expertise to confirm that it is of an acceptable scientific standard.

# Peer review: Reviewer response for version 1

**DOI:** 10.21956/wellcomeopenres.17912.r40691

**Published:** 2020-10-09

**Authors:** Ciro Chiappini

**Affiliations:** 1 Centre for Craniofacial and Regenerative Biology, King’s College London, London, UK; 2 London Centre for Nanotechnology, London, UK

The article provides an interesting and insightful overview of the existing and emerging approaches to harvesting mitochondria from cells and tissues. The topic is highly timely and of great interest given recent advances in our ability to extract, manipulate and transfer mitochondria which promise significant improvements in mithocondria-based therapies in the coming future.

 The different available and emerging approaches are discussed in sufficient length and well compared, with clear and useful tables to highlight and compare their relative strengths and limitations.

 The focus on the microscale and nanoscale isolation techniques is particularly useful, providing a clear overview of these emerging technologies, their state of advancement and their potential for the future.

 Only minor suggestions are made on one side to improve the readability of the article and on the other side to provide further insight into future developments and challenges for the emerging techniques reviewed.

1. The abstract can be reformulated to improve its flow. "Whilst" should not be followed by a comma. In the same sentence, due to the structure, it's unclear whether single cell technologies refers to mitochondrial isolation technologies or broadly single-cell analysis technologies, and it makes a significant difference if one or the other. Reformulating the entire sentence could help a more unambiguous interpretation. The final sentence is also fairly complex and requires careful reading to extract the correct meaning. Possibly splitting it into two would help a more direct interpretation. Particularly the - from subcellular compartment - interjection is of difficult placement.
2. The sentence "due to nuclear mutations that lead to mtDNA mutation formation throughout life." would benefit from clarification. Is it meant due to nuclear [genetic material/DNA] mutations? Otherwise what other mutations are involved? also, maybe worth adding an explanatory sentence regarding how the nuclear mutations lead to mtDNA mutation (and possibly heterogeneity).
3. The term OXPHOS is not defined before it is introduced.
4. CE-LIF acronym is not defined before introducing. only CE is defined.
5. In Table 1 the lower-and/or higher sign (plus-minus) does not appear to be used in the table, so it would be better to removed from the caption as well.
6. Please expand on the following statement by indicating the approach to achieve superior positional placement through SICM in a practical system involving mitochondrial extraction. "positional placement using an SICM system guided by topographical scanning rather that just optical confirmation alone, would suggest that Nano biopsy would have a superior sampling specificity (Seger *et al.*, 2012)."
7. Please expand on the following statement by indicating the logistics of integrating
  SICM with large pipettes and the contrast to conventional micromanipulator-operated micropipettes. Interestingly, since mitochondria can be as large 2µm in diameter (Imura *et al*., 2002), incorporation of micropipettes within an SICM system may enable nanoscale precision but also guards against sampling bias favouring smaller mitochondrial subpopulations.
8. It would be very valuable either in the concluding remarks to discuss key early potential applications for nano-isolation techniques, their range of applicability, the limits of such range and the outstanding challenges to achieve the first practical uses. e.g.. in vivo? What downstream analysis would this approach be compatible with? What are the challenges associated with the need to investigate intracellular heterogeneity which by definition would employ very limited amounts of biological materials, and what emerging approaches in the analytical field are emerging to address them? What avenues are accessible to increase yield/purity/throughput for these technologies?
9. Further insightful discussions for the conclusion sections would be: In the context of the omics discussion already presented, what could be an avenue for integration of these technologies? Mitochondrial transfer applications are highly exciting. What are potential strategies to achieve them using nano approaches? What barriers still exist to prove the feasibility of mitochondrial injection with a nanoprobe?

Is the review written in accessible language?

Yes

Are all factual statements correct and adequately supported by citations?

Yes

Are the conclusions drawn appropriate in the context of the current research literature?

Yes

Is the topic of the review discussed comprehensively in the context of the current literature?

Yes

Reviewer Expertise:

Nanomedicine

I confirm that I have read this submission and believe that I have an appropriate level of expertise to confirm that it is of an acceptable scientific standard.
